# HER2-low metastases of HER2-negative primary tumors: a single institution analysis of intertumoral and internodal heterogeneity in node-positive breast cancer

**DOI:** 10.3389/fonc.2023.1167567

**Published:** 2023-07-07

**Authors:** Ulrika Pellas, Annette Bauer, Ilija Vladimir Baroš, Caterina Fattorini, Tibor Tot

**Affiliations:** ^1^ Unit for Research and Higher Education, Centre for Clinical Research Dalarna, Uppsala University, Region Dalarna, Falun, Sweden; ^2^ Pathology and Cytology Dalarna, County Hospital Falun, Region Dalarna, Falun, Sweden; ^3^ College of Health Sciences, Pan-European University, Banja Luka, Bosnia and Herzegovina; ^4^ Pathology Unit, Azienda Sanitaria Toscana Nord-Ovest, Pisa, Italy

**Keywords:** breast cancer, human epidermal growth factor receptor 2, HER2, heterogeneity, HER2-low, lymph node, metastasis, Gene-Protein Assay (GPA)

## Abstract

**Objective:**

HER2 status in breast cancer is an essential parameter in individual therapeutic decision-making and is routinely assessed in primary tumors in accordance with international recommendations. Reports of HER2 heterogeneity raise the question of basing treatment decisions on HER2 status in metastases, if present. We investigated the degree and clinical implications of HER2 heterogeneity in lymph node–positive breast cancer. Because of recent recognition of therapeutic opportunities in this group of tumors, we especially focused on cases involving low-level HER2 expression.

**Methods:**

The HER2 status of primary tumors and of corresponding lymph node metastases was determined in archived material at the protein and gene levels using the gene– protein assay and interpreted in accordance with 2018 ASCO/CAP criteria. HER2-low status was defined as protein expression levels 1+ or 2+ with negative amplification status.

**Results:**

We analyzed a series of 43 cases of primary infiltrating breast cancer, each with at least two axillary nodes harboring macrometastases (>2 mm), in total 206 such nodes. In 7% of cases, we detected intertumoral HER2 heterogeneity. Three of nine HER2-positive primary tumors were associated with HER2-negative metastases. No cases with HER2-negative primary tumors had HER2-positive metastases, but 55% (6/11) of HER2 0 primary tumors had HER2 1+ and/or 2+ metastases, and 19% (3/16) HER2 1+ cases had exclusively HER2 0 metastases. All metastases in HER2 2+ cases showed HER2-low protein expression levels. Internodal HER2 heterogeneity at low protein expression levels (presence of HER2 0, HER2 1+, and/or HER2 2+ metastatic deposits within the same axilla) was seen in 40% (17/43) of cases. We found no statistically significant association between HER2 heterogeneity and other tumor-related parameters. Survival data indicated worse outcomes in the HER2-low group compared with the rest of the cohort.

**Conclusion:**

Our results indicate a substantial instability of HER2 protein expression, leading to considerable intertumoral and internodal HER2 heterogeneity in lymph node–positive breast carcinomas. This heterogeneity is particularly relevant in HER2-low tumors in which the corrective effects of HER2 gene copy number analysis definitionally is absent. Our findings suggest that determining HER2 status in metastatic lymph nodes may generate relevant information for therapeutic decision-making.

## 1 Introduction

Breast cancer is a heterogeneous disease (1), divided into several distinct subtypes. Expression levels of tumor biomarkers such as estrogen receptor (ER), progesterone receptor (PR), and human epidermal growth factor receptor 2 (HER2) are pivotal for treatment decisions and prognostication in breast cancer. Approximately 15%–20% of all breast cancers are HER2-positive, attributable to over-expression of the HER2 protein and/or increased copy number of the HER2 gene (amplification) (2). Findings suggest that expression levels of HER2 might vary within a single tumor focus (intratumoral heterogeneity) as well as between different tumor foci and between the primary tumor(s) and synchronous axillary lymph node metastases (intertumoral heterogeneity) (3–5). HER2 heterogeneity is a major challenge for accurate evaluation of HER2 status and affects both prognosis and treatment success (6–12). In clinical practice, prognosis and treatment decisions are regularly based on the evaluation of biomarkers in primary tumors, even in cases involving synchronous axillary metastases.

The group of HER2-low breast carcinomas represents a newly defined entity in the field, thanks to the observation that treatment with trastuzumab duocarmazine led to partial response in 28% and 40% of patients with HER2-low ER-positive and ER-negative breast cancer, respectively (13). This finding highlighted the need to delineate the group of patients who might benefit from a targeted therapy because the target is present in some tumor cells even in the absence of the amplified oncogene. The HER2-low category comprises a spectrum of carcinomas with levels of protein expression up to HER2 2+. These tumors are associated with a poorer prognosis than with HER2-negative cancers (14–16).

We previously showed that HER status may differ between the primary tumor and metastases in as many as 13.2% of cases (12). We also observed internodal HER2 heterogeneity of uncertain significance in our cases, which suggested the need for more studies with an expanded panel of metastases (12). Sapino et al. reported a high prevalence of HER2 heterogeneity in HER2-low tumors (17), which motivated us to focus our study on this tumor category.

## 2 Materials and methods

### 2.1 Breast cancer cases

We retrospectively identified patients diagnosed with primary invasive breast cancer at the Department of Pathology and Cytology Dalarna of the County Hospital Falun in Sweden between 2011 and 2015. To allow for analysis of internodal heterogeneity, only cases involving at least two axillary lymph node macrometastases were included, with macrometastasis defined as a metastatic deposit >2 mm within a lymph node (18). Patients were included if they had provided informed consent at the time of diagnosis, allowing us to use parts of their archived tumor material for this study. Patients with recurrent disease and those who received neoadjuvant treatment before surgery were excluded. Availability of tumor and metastatic material was a requirement for inclusion. This study was approved by the ethical review board in Uppsala, Sweden (registration number 2010/461, 2010/461/1), and by the Swedish Ethical Review Authority (registration number 2020-00310).

### 2.2 Tumor material

Clinico-pathological parameters such as tumor size, disease extent, growth pattern/lesion distribution, TNM stage, Nottingham histology grade, and biomarker profile/molecular phenotype were retrieved from the medical records (for the definitions of these parameters, see reference (19)). An experienced breast pathologist reexamined the primary tumors of the selected cases. Formalin-fixed, paraffin-embedded archived material from the metastatic lymph nodes was retrieved for this work. Sections were cut in 4 µm slices from the most representative paraffin blocks and stained as described below.

### 2.3 Gene–protein assay (GPA)

Newly sliced tumor material from metastatic lymph nodes was stained using the Roche GPA method, as previously described (20), with primary tumors re-stained only in cases for which GPA was unavailable from the time of diagnosis. In brief, the HER2 protein was stained using an HER2/neu rabbit monoclonal primary antibody (clone 4B5, Ventana Medical Systems, Inc., Tucson, AZ, USA). A dual chromogen *in situ* hybridization (ISH) was performed on this material to quantitatively detect the HER2 gene as well as the chromosome 17 centromere (CEN17). Silver ISH (SISH) was used to visualize the HER2 gene and chromogen red ISH (Red ISH) to visualize CEN17. The slides were counterstained with hematoxylin II and bluing reagent. These assays were performed using the BenchMark^®^ XT (Ventana Medical Systems, Inc.), with NexES software and the INFORM HER2 Dual ISH DNA Probe Cocktail mix (Ventana Medical Systems, Inc.) in accordance with the manufacturer’s recommendation.

The stained slides were analyzed using a brightfield microscope (Olympus BX45) for HER2 status using a 60× objective. At least three separate distant foci of the tumor cells in a single metastatic lymph node were analyzed for HER2. Two experienced pathologists performed the analyses without awareness of the patient’s clinical status. HER2 status was assessed in 120 tumor cells (40 cells per focus) according to the 2018 ASCO/CAP guidelines (21).

Status was assessed as HER2-positive (1) if the immunohistochemical staining was rated 3+; (2) if the immunohistochemical staining was rated 2+, the HER2/CEP17 ratio was ≥2, and the average number of copies of the HER2 gene was >4.0; or (3) if the immunohistochemical staining was rated 2+, the HER2/CEP17 ratio was <2, and the average number of copies of the HER2 gene was ≥6.0. Status was assessed as HER2-negative if the tumor did not fulfill the above criteria. Primary tumors and metastases were defined as HER2-low if protein expression levels were rated HER21+ or HER2 2+ and were non- amplified (i. e., the HER2/CEP17 ratio was <2 and/or the average number of copies of the HER2 gene was ≤4.0). Tumors and metastases with HER2 protein expression level 0 were regarded as HER2-negative.

Intertumoral heterogeneity was defined as any discrepancy in HER2 protein expression levels and/or gene amplification status between the primary tumor and its lymph node metastases. Internodal heterogeneity was defined as the same discrepancy in HER2 levels being present between the metastases located to lymph nodes within the same axilla.

### 2.4 Statistical analysis

Numerical data were presented as means, standard deviations, and ranges. Categorical data were presented as counts (n) and proportions. Analysis of the statistical significance was performed using the independent samples t-test (student's t-test) or Fisher's exact test. A statistical result was considered significant when P < 0.05. Statistical analysis was performed using Jamovi software (22, 23). The potential impact of the results on prognosis and therapeutic decisions was estimated based on Swedish national recommendations (24).

## 3 Results

All included patients were women, with a mean age of 59.8 years. Extensive tumors (e.g., >40 mm) were found in 29 (67.4%) of the cases. The aggregate growth pattern was unifocal in 13 (30.2%), multifocal in 13 (30.2%), and diffuse in 17 (39.5%) of the cases. Of the 43 included cases, 10 (23.3%) were luminal A-like, 22 (51.2%) were luminal B-like, and 2 (4.6%) were triple negative, while 9 (20.9%) were HER2-overexpressing. The number of macrometastases in the ipsilateral lymph nodes ranged from 2 to 23. In total, 206 macrometastases and 43 primary tumors were analyzed for HER2 status. Details of the clinico-pathological parameters in this cohort are shown in **Table 1**.

**Table 1:** Patient characteristics and pathological parameters of the primary tumor by HER2 status.

HER2 status according to ASCO/CAP 2018							
		HER2 negative (n = 34)	HER2 positive (n = 9)	*P*	HER2 0 (n = 11)	HER2-low (n = 23)	*P*
	Mean (±SD)	59.8 (±11.6)	59.8 (±12.1)	0.997*	56.5 (±5.6)	61.4 (±13.4)	
Age at diagnosis							0.251*
	Mean (±SD)	4.9 (±4.4)	4.4 (±3.5)	0.783*	5.9 (±6.6)	4.4 (±2.8)	
Number of lymph nodes containing macrometastases							0.352*
	Range	2–23	2–13		2–23	2–12	
Tumor size (mm)	Mean (±SD)	31.8 (±20.4)	40.1 (±18.5)	0.274*	32.1 (±22.0)	31.6 (±20.2)	0.950*
	Range	10–100	12–78		14–90	10–100	
Total extent, number of cases n (%)	Extensive (≥40 mm)	22 (64.7)	7 (77.8)	0.693^†^	8 (72.7)	14 (60.9)	0.705^†^
	Non- extensive	12 (35.3)	2 (22.2)		3 (27.3)	9 (39.1)	
Collective growth pattern, number of cases, n (%)	Unifocal	11 (32.4)	2 (22.2)	0.579^†^	2 (18.2)	9 (39.1)	0.218^†^
	Multifocal	11 (32.4)	2 (22.2)		6 (54.5)	5 (21.7)	
	Diffuse	12 (35.3)	5 (55.6)		3 (27.3)	9 (39.1)	
Tumor grade, number of cases, n (%)	Grade I	3 (8.8)	0 (0.0)	0.724^†^	1 (9.1)	2 (8.7)	0.734^†^
	Grade II	21 (61.8)	5 (55.6)		8 (72.7)	13 (56.5)	
	Grade III	10 (29.4)	4 (44.4)		2 (18.2)	8 (34.8)	
Molecular phenotype, number of cases, n (%)	Luminal A- like	10 (29.4)	0 (0.0)	<0.001^†^	4 (36.4)	6 (26.1)	0.598^†^
	Luminal B- like	22 (64.7)	0 (0.0)		6 (54.5)	16 (69.6)	
	Triple negative	2 (5.9)	0 (0.0)		1 (9.1)	1 (4.3)	
	HER2	0 (0.0)	9 (100.0)		0 (0.0)	0 (0.0)	

*Calculated using independent samples t-tests (student’s t-test).

^†^Calculated using Fisher’s exact test.

### 3.1 HER2 status according to the 2018 ASCO/CAP criteria

**Figure 1:**
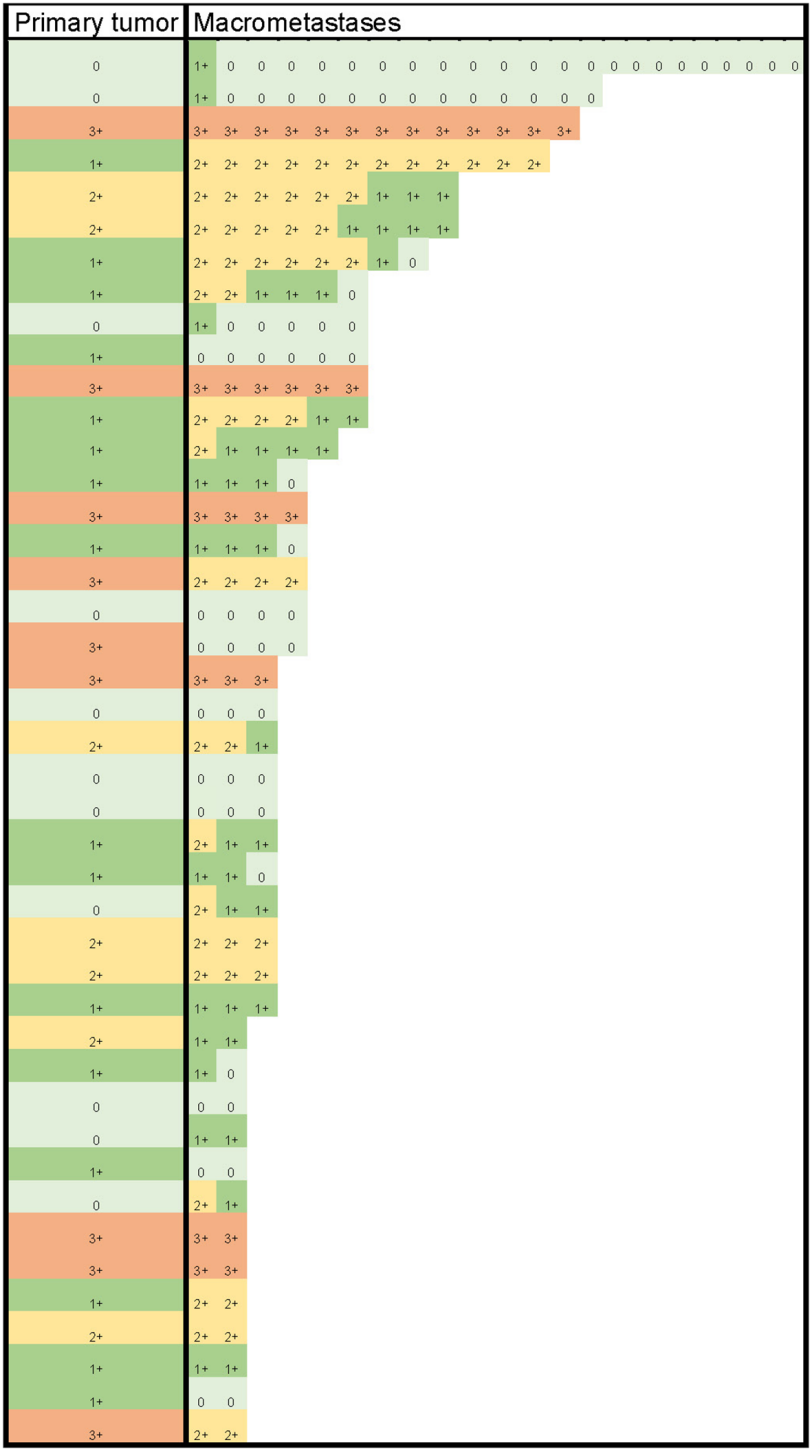
HER2 protein levels (color-coded) in primary tumors and their macrometastases.

HER2 status (negative/positive) was determined based on HER2 protein levels, HER2/CEP17 ratio, and average HER2 copy number, according to the 2018 ASCO/CAP guidelines (21). None of the 34 cases with a primary tumor diagnosed as HER2-negative displayed HER2-positive macrometastases. Of the nine cases with HER2-positive primary tumors, three (33.3%) had HER2-negative macrometastases, with no signs of internodal heterogeneity among the positive lymph nodes. The nine cases with HER2-positive primary tumors collectively had 30 (75.0%) HER2-positive and 10 (25.0%) HER2-negative macrometastases. The details from the comparison of HER2 levels in primary tumors with those in macrometastases are presented in **Table 2** and **Figure 1**.

**Table 2:** HER2 status of primary tumors compared to HER2 status in macrometastases in ipsilateral lymph nodes.

Metastases in the ipsilateral lymph node Number of cases, n (%)					
Primary tumor	Protein level	0/1+	2+	3+	Total
	0/1+	25 (92.6)	9 (33.3)	0 (0.0)	27a (62.8)
	2+	4 (57.1)	6 (85.7)	0 (0.0)	7b (16.3)
	3+	1 (11.1)	2 (22.2)	6 (66.7)	9 (20.9)
	43				
	HER2/CEP17 ratio	<2		≥2	
	<2	31 (96.9)		1 (3.1)	32 (76.2)
	≥2	2 (20.0)		10 (100)	10c (23.8)
					42d
	Average HER2 copy number (signals/cell)	<4.0		≥4.0	
	<4.0	34 (100.0)		0 (0.0)	34 (81.0)
	≥4.0	3 (37.5)		5 (62.5)	8 (19.0)
					42d
	HER2 statuse	Negative		Positive	
	Negative	34 (100)		0 (0.0)	34 (79.1)
	Positive	3 (33.3)		6 (66.7)	9 (20.9)
	43				

a) Seven cases with HER2 0/1+ primary tumors displayed both macrometastases diagnosed as HER2 0/1+ and macrometastases diagnosed as HER2 2+.

b) Three cases with a primary tumor diagnosed as HER2 2+ showed both macrometastases diagnosed as HER2 0/1+ and macrometastases diagnosed as HER2 2+.

c) Two cases with a primary tumor showing a HER2/CEP17 ratio ≥2 displayed both macrometastases with a HER2/CEP17 ratio <2 and macrometastases with a HER2/CEP17 ratio ≥2.

d) One case including one primary tumor and two macrometastases was excluded from the analysis because the primary tumor was not genetically analyzed.

e) Based on HER2 protein level, HER2/CEP17 ratio, and average HER2 copy number, in accordance with the 2018 ASCO/CAP guidelines.

### 3.2 Intertumoral and internodal heterogeneity in HER2-low tumors

Of the cases with HER2-negative (HER2 0) primary tumors, 55% (6/11) had macrometastases with HER2 1+ and/or 2+ protein expression levels in at least one positive lymph node, qualifying the metastases as being HER2-low. In 19% (3/16) of cases, HER2 1+ primary tumors lost their protein expression, becoming HER2 0 in all of their metastatic deposits. All seven HER2 2+ primary tumors had HER2-low lymph node deposits. Internodal HER2 heterogeneity was found at the protein level in 40% (17/43) of cases, as follows: in seven cases with both HER2 0 and HER2 1+ metastases, in eight cases with both HER2 1+ and 2+, and in two cases with HER2 0, 1+, and 2+ metastases within the same axilla. None of the axillae simultaneously contained HER2-low and HER2 3+ tumor deposits. The details of the comparisons of HER2 in macrometastases to HER2 in the primary tumor are shown in **Table 3** and **Figure 1**.

**Table 3:** Results from comparing HER2 levels in primary tumors and macrometastases with regard to HER2-low.

Metastases in the ipsilateral lymph node Number of cases, n (%)						
	HER2 protein levels	0	1+	2+	3+	Total
Primary tumor					
	0	8 (72.7)	6 (54.5)	2 (18.2)	0 (0.0)	11a
	1+	9 (56.3)	11 (68.8)	7 (43.8)	0 (0.0)	16b
	2+	0 (0.0)	4 (57.1)	6 (85.7)	0 (0.0)	7c
	3+	1 (11.1)	0 (0.0)	2 (22.2)	6 (66.7)	9
	43					
	Number of metastases, n (%)					
	HER2d	Negative	Low		Positive	Total
	Negative	55 (84.6)	10 (15.4)		0 (0.0)	
						65
	Low	16 (15.8)	85 (84.2)		0 (0.0)	
						101
	Positive	4 (10.0)	6 (15.0)		30 (75.0)	
						40
	206					

	HER2 protein levels	0	1+	2+	3+	
	0	55 (84.6)	8 (12.3)	2 (3.1)	0 (0.0)	
						65
	1+	16 (22.9)	26 (37.1)	28 (40.0)	0 (0.0)	
						70
	2+	0 (0.0)	10 (32.3)	21 (67.7)	0 (0.0)	
						31
	3+	4 (10.0)	0 (0.0)	6 (15.0)	30 (75.0)	
						40
	206					

a) Two cases with HER2 0 primary tumors had both macrometastases that were HER2 1+ and macrometastases that were 2+. Three cases with HER2 0 primary tumors had both macrometastases that were HER2 1+ and macrometastases that were 0.

b) Four cases with HER2 1+ primary tumors had both macrometastases that were HER2 1+ and macrometastases that were 0. Three cases with HER2 1+ primary tumors had both macrometastases that were HER2 1+ and macrometastases that were 2+. Two cases with HER2 1+ primary tumors had macrometastases that were HER2 1+, macrometastases that were 0, and macrometastases that were 2+.

c) Three cases with HER2 2+ primary tumors had both macrometastases that were HER2 1+ and macrometastases that were 2+.

d) Negative: HER2 0. Low: HER2 1+ or 2+ and not amplified, i.e., HER2/CEP7 ratio <2 or HER2/CEP17 ratio ≥2 and an average HER2 copy number <4.0. Positive: HER2 3+.

### 3.3 Breast cancer–specific survival data

Of the 43 patients represented in this work, 10 died of breast cancer during the observation period up to 13 October 2021, half of them within 5 years after diagnosis. The survival time of the 10 patients who died varied from 1202 to 3626 days (mean, 2196 days). No statistically significant differences were found between surviving group and the group that died of the disease when comparing their ER and PR status, tumor size, pathological T-stage and N-stage and number of metastatic lymph nodes (**Supplementary Table S1**). The average number of positive nodes among the fatal cases was 13.6 (41/3) in the HER2 0 group, 3.7 (60/16) in the HER2 1+ group, and 4.4 (31/7) in the HER2 2+ group. None of the patients included in this studies had distant metastases at the time of diagnosis. None of the patients with HER2-positive primary tumors died of the disease during this period. Three of eleven patients with HER2-negative primary tumors (HER2 0) had a fatal outcome, as did 7 of 23 patients with tumors showing low HER2 protein expression levels (4/16 HER2 1+, 3/7 HER2 2+). Two of the three HER2 0 patients with a fatal outcome had a HER2 1+ lymph node deposit in one of their metastases, so that all but one of the fatal cases involved HER2-low protein expression levels in the primary or metastatic tumors or both.

## 4 Discussion

In the present study 37% (16/43) of the cases had at least one metastasis with HER2 protein levels diverse from those in the primary tumor. This finding is in line with results of previously published studies. A review of similar studies comparing HER2 protein expression in primary tumors and metastases described discordances of up to 33.2% (25). Other studies, however, have shown a high concordance of HER2 status between primary tumor and metastases, with discrepancy rates as low as 2% (26) and 3.4% (4). Analysis of HER2 status at the gene level in our study showed markedly higher concordance than results at the protein level, with only 7% (3/43) of cases (i.e., 3 HER2-positive tumors) losing their HER2-positive status in metastases. Cho et al. reported similar findings, reporting a discordance of 18% in protein expression levels and 6% with chromogenic ISH when comparing the HER2 status of primary breast cancer and paired metastatic lymph nodes (27). The same tendency was seen in the study showing a 3.4% rate of discordant protein expression but concordant gene amplification status in all cases (4). Another study using FISH demonstrated concordant HER2 amplification status between the primary tumor and synchronous axillary metastases in all analyzed cases (3).

The HER2-low tumor category is a newly defined group of breast carcinomas that express low levels of HER2 protein in the membrane of the tumor cells up to HER2 2+ level but show no amplification of the HER2 gene (28, 29). These tumors may partially respond to modern anti-HER2 treatment, and their delineation from HER2-negative breast carcinomas is essential (12) because these patients may potentially benefit from anti-HER2 targeted therapy. However, delineating HER2 1+ carcinomas from HER2 0 tumors is a real challenge in everyday practice, and intratumoral heterogeneity, intraobserver and interobserver variations, and technical issues represent the main obstacles (29). To avoid these potential technical pitfalls, we reassessed the HER2 slides of the primary tumors and stained the sections from the metastases in batches in the same instrument, using identical protocols and reagent kits. By analyzing 120 cells (i.e., three times the recommended 40 cells), we tried to eliminate the influence of intratumoral heterogeneity. Analysis was done by two experienced pathologists unaware of the patient’s clinical status. The GPA method is particularly useful in assessing the HER2 status of heterogeneous tumors (20) and in our case, also in assessing HER2-low status.

As noted, HER2 protein expression levels may vary in a considerable proportion of breast carcinomas, and HER2-low tumors, defined by their protein expression levels, are known for their intratumoral heterogeneity (11). In addition, HER2-low heterogeneity is evident in a high proportion of multifocal cancers, between the multiple simultaneous invasive tumors within the same breast (11). Our study also demonstrated a substantial intertumoral heterogeneity in HER2-low tumors, with discrepancies in HER2 protein expression levels between the primary tumor and its metastases in a third of HER2-negative primary tumor cases (HER2 0, 1+, and 2+, non-amplified). We found that 55% (6/11) of HER2 0 tumors gained HER2-low status (HER2 1+ and/or 2+) in at least one lymph node metastasis and 19% (3/16) of HER2-low (HER2 1+) cases lost protein expression, showing HER2 0 expression in all metastases. All HER2 2+ primary tumors had HER2-low lymph node deposits.

Discordance in HER2 status between two or more metastatic foci has been more sparsely studied. Gancberg et al. reported an 18% discordance in different metastatic sites (30). We analyzed cases with two or more macrometastases in the ipsilateral lymph nodes and showed that lymph node metastases displaying diverse HER2 protein levels were common, seen in 40% of our HER2-low cases.

We found no statistically significant association between HER2-low tumor status and age, histological tumor grade, molecular tumor phenotype, tumor size, disease extent, or lesion distribution. In contrast, Baez-Navarro et al. found in a much larger cohort that HER2-low tumors were significantly associated with histologic subtype, a higher ER, and lower PR expression in their ER+ cohort, whereas within the ER- cohort, HER2-low tumors were associated with a lower tumor grade (11).

HER2-low tumors seem to carry a poorer prognosis compared with HER2-negative cancers (14–16). Our results also indicate that HER2-low status impacts survival, with 9 of 10 fatal outcomes in this study occurring in the group showing HER2-low primary tumors, HER2-low metastases, or both. The patients with fatal HER2-negative (HER2 0) primary tumors had a much larger metastatic tumor burden than those with fatal HER2-low tumors, which may explain the fatal outcome in HER2 0 cases. No HER2-positive patients died of the disease in our study, which may be related to efficient targeted therapy. The large number of fatal HER2-low cases may be attributable to the fact that these patients, in accordance with international and national recommendations during the studied period, did not receive anti-HER2 therapy. A recent large retrospective study with a limited follow-up time showed no evidence of significant differences in overall survival associated with HER2-low and HER2-0 tumors (11).

Weaknesses of this study include its retrospective character and the limited number of patients included after application of rigorous selection criteria. These limitations preclude conclusions regarding potential differences between the clinicopathological parameters in the HER2 subgroups.

## 5 Conclusions

Our results demonstrate a substantial instability of HER2 protein expression leading to considerable intratumoral and internodal HER2 heterogeneity in lymph node–positive breast carcinomas. This finding is particularly relevant for HER2-low tumors which definitionally lack the corrective effects of HER2 gene copy number analysis. Although the clinical impact of these findings remains unclear and warrants larger studies, our results suggest that determining HER2 status in metastatic lymph nodes may generate relevant information for therapeutic decision-making.

## Data availability statement

The raw data supporting the conclusions of this article will be made available by the authors, without undue reservation.

## Ethics statement

The study involving human participants was reviewed and approved by The Ethical Committee of the Uppsala–Örebro Region in Sweden, EPN No 2010/461, and the Swedish Ethical Review Authority, No 2020-00310 and 2019-01739. The patients/participants provided their written informed consent to participate in this study.

## Author contributions

Conceptualization: UP and TT; Methodology: UP, TT, and AB; Material preparation and data collection: UP, CF, IB, AB, and TT; Formal analysis and investigation: AB, CF, IB, and UP; Writing - original draft preparation: UP and TT; Writing - review and editing: all authors; Acquisition of funding: UP and TT. All authors contributed to the article and approved the submitted version.
