# Supplementary material for: HER2-low metastases of HER2-negative primary tumors: a single institution analysis of intertumoral and internodal heterogeneity in node-positive breast cancer
Source: Front Oncol. 2023 Jul 7;13:1167567. doi: 10.3389/fonc.2023.1167567 (PMC10362429; doi:10.3389/fonc.2023.1167567)
Supplement: Supplementary file 1 [file Table_1.docx]

|  |  | | | | | | |
| --- | --- | --- | --- | --- | --- | --- | --- |
|  |  | Survived total follow up period  (n = 32) | Dead within follow up period  (n = 10) | *P* | Survived >5 years after diagnosis  (n = 37) | Dead within 5 years after diagnosis  (n = 5) | *P* |
| Number of metastases located to ipsilateral lymph node | Mean (±SD)  Range | 4.4  (±3.1)  2-14 | 8.0  (±8.8)  2-29 | 0.053* | 4.9  (±5.0)  2–29 | 8.0  (±6.1)  2–15 | 0.21* |
| N-staging, number of cases  n (%) | pN1 | 17  (53) | 5  (50) | 0.252^†^ | 20  (54) | 2  (40) | 0.316^†^ |
|  | pN2 | 12  (38) | 2  (20) |  | 13  (35) | 1  (20) |  |
|  | pN3 | 3  (9) | 3  (30) |  | 4  (11) | 2  (40) |  |
| Tumor size (mm) | Mean (±SD) | 31.4  (±16.4) | 41.0  (±29.6) | 0.198* | 34.8  (±21.2) | 26.0  (±11.0) | 0.198* |
|  | Range | 10–78 | 14–100 |  | 10–100 | 14–40 |  |
| T-staging, number of cases  n (%) | pT1 | 10  (31) | 2  (20) | 0.691^†^ | 10  (27) | 2  (40) | 0.829^†^ |
|  | pT2 | 18  (56) | 6  (60) |  | 21  (57) | 3  (60) |  |
|  | pT3 | 4  (13) | 2  (20) |  | 6  (16) | 0  (0) |  |
| ER status,  number of cases  n (%) | Negative | 3  (9) | 1  (10) | 1.00^†^ | 3  (8) | 1  (20) | 0.41^†^ |
|  | Positive | 29  (91) | 9  (90) |  | 34  (92) | 4  (80) |  |
| PR status,  number of cases,  n (%) | Negative | 7  (22) | 5  (50) | 0.117^†^ | 9  (24) | 3  (60) | 0.131^†^ |
|  | Positive | 25  (78) | 5  (50) |  | 28  (76) | 2  (40) |  |

*P-value calculated using Independent Samples T-Test, Student´s t

^†^ P-value calculated using Fisher`s exact test
